# Parental Obesity Predisposition and Age of Onset Associate with Poor Response to Bariatric and Metabolic Surgery

**DOI:** 10.1007/s11695-023-06499-1

**Published:** 2023-03-01

**Authors:** Mira Fink, Stephan Herrmann, Jodok Fink, Claudia Lässle, Goran Marjanovic, Luca Fagnocchi, J. Andrew Pospisilik, Stefan Fichtner-Feigl, Gabriel Seifert

**Affiliations:** 1 Department of General and Visceral Surgery, Medical Center Freiburg, University of Freiburg, Hugstetter Strasse 55, 79106 Freiburg, Germany; 2 Department of Epigenetics, Van Andel Institute, 333 Bostwick Ave NE, Grand Rapids, MI 49503 USA

**Keywords:** Bariatric and metabolic surgery, Surgical weight loss failure, Familial predisposition, Onset of obesity

## Abstract

**Introduction:**

Parental predisposition and age of onset may be independently associated with 1-year total weight loss (TWL) failure (< 20%) after metabolic–bariatric surgery (MBS).

**Methods:**

This cohort study includes all cases of the German StuDoQ|MBE register (2015–2019) with data on parental predisposition, obesity onset, and at least 1-year follow up after primary MBS procedures (*n* = 14,404). We provide descriptive statistics of the cohort in terms of the main outcome and 1-year TWL failure, and provide characteristics of surgery type subgroups. Finally, we provide a multivariate logistic regression model of 1-year TWL failure.

**Results:**

58.8% and 45.7% of patients reported maternal and paternal predisposition for obesity, respectively. Average onset of obesity was 15.5 years and duration of disease 28.3 years prior to MBS. SG is the most frequently performed procedure (47.2%) followed by RYGB (39.7%) and OAGB (13.1%). Mean 1-year TWL is 32.7 ± 9.3%, and 7.8% (*n* = 1,119) of patients show TWL failure (< 20%). Multivariate analysis shows independent association of early onset of obesity (< 18 years), male sex, age at operation, pre-operative BMI, pre-operative weight loss, sleeve gastrectomy (SG), and type 2 diabetes (T2D) with 1-year TWL failure (*p* < 0.001).

**Conclusion:**

The proportions of MBS patients that report on paternal and maternal predisposition for obesity are 45.7% and 58.8% respectively, and average age at onset is 15.5 years. 7.8% of patients do not meet current target criteria of successful response to surgery at 1 year. Early onset, male sex, age at operation, pre-operative BMI, pre-operative weight loss, SG, and T2D are independently associated with weight loss failure.

**Graphical abstract:**

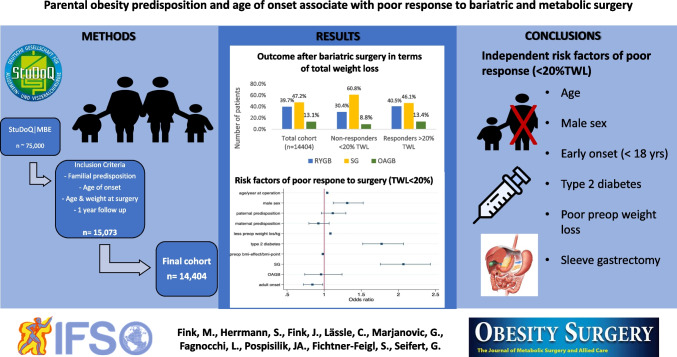

## Introduction

Metabolic-bariatric surgery (MBS) remains the most effective long-term treatment for morbid obesity [1]. However, individual response to standardized surgical procedures varies significantly [2]. A relevant proportion of patients (up to 11%) do not achieve adequate total weight loss (TWL) within 1 year of MBS [3, 4]. Weight loss < 20% is commonly defined as “surgical weight loss failure”, patients with weight loss failure as “non-responders”. Identification of relevant clinical risk factors associated with TWL failure and mechanisms underpinning poor response are important areas of research [5–10].

In this study, we investigate the association of parental predisposition and onset of disease with 1-year TWL failure. We describe the distribution of parental predisposition and age at onset in surgical 1-year TWL success versus failure in a cohort of 14,404 MBS patients of the German StuDoQ|MBE register. Secondly, we describe current clinical profiles and outcomes of the most common types of surgery, Roux-en-y gastric bypass (RYGB), sleeve gastrectomy (SG), and one-anastomosis gastric bypass (OAGB). Finally, we model independent predictors of 1-year TWL failure, adjusting for parental predisposition and onset of obesity.

## Methods

### Patient Selection

**Fig. 1:**
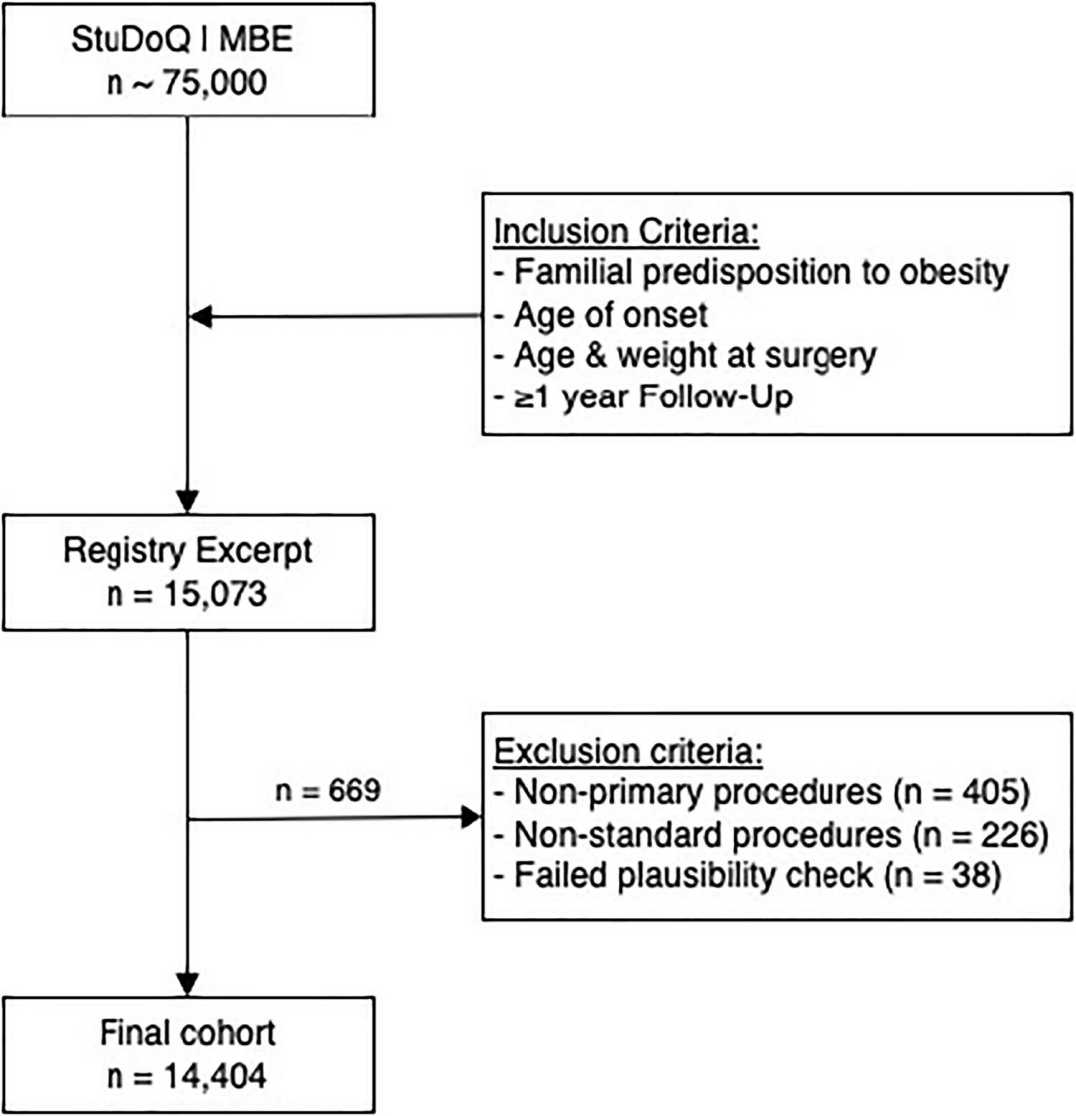
Flow chart of cohort selection. Of ~ 75,000 observations in the StuDoQ | MBE register at the time of application, a search was conducted with the primary inclusion criteria “familial predisposition to obesity,” “age of onset,” “age & weight at surgery,” “ > follow-up ≥ 1 year.” There were 15,073 cases identified. We excluded non-primary procedures, non-standard and rare procedures as well as observations that failed a plausibility check during quality control of the data. The final study cohort entailed 14,404 observations

Since 2015, StuDoQ|MBE’s database serves continuous monitoring, improvement of clinical practice and facilitation of clinical research in MBS patients. The register contains prospectively collected data on more than 75,000 patients, all of which provided signed informed consent. After a formal application, data was exported in August 2020 and included all patients with information on familial predisposition of obesity, age of onset, and at least 1-year follow-up (*n* = 15,073). Primary SG, RYGB, and OAGB were included, and revisional or re-do surgery and rare, non-standard, and combined procedures were excluded (*n* = 631). After quality control and exclusion of unfeasible observations, the final number of patients included in this study was *n* = 14,404 (Fig. 1).

### Statistical Analysis

We used SPSS Version 22 (SPSS; 2016) and STATA Version 17 for statistical investigation. 1-year TWL was chosen as the primary endpoint of our descriptive analyses, and non-response was defined as TWL < 20%. 1-year failure was modelled in a multi-variate regression analysis. Sample size at 3 years was too small to model independently. We describe continuous data as mean values ± standard deviation and dichotomous data as proportions. After exploring crude univariate associations of pre-operative covariates with 1-year TWL failure, we fitted a multivariate logistic regression model to estimate their adjusted association with the primary endpoint. For this model, “unknown” parental predisposition for obesity was treated as “missing.” We conducted a complete case analysis. The model includes pre-operative clinical factors including parental predisposition and onset of obesity. We performed a post-estimation test according to Hosmer–Lemeshow to investigate goodness of fit. The odds ratio coefficients of this model were plotted using coefplot in STATA [11]. Discriminatory power of the model was tested by calculating a receiver operating characteristic (ROC) curve of the final model. We used the DeLong method to compare ROC-curves of two multi-variate models to estimate the relevance of adding surgery type as a pre-operative predictor of TWL failure. A *p*-value < 0.05 was considered statistically significant.

## Results

### Cohort Characteristics

We included 14,404 patients in this study. 26.1% of patients were male (*n* = 3756). Mean age was 44 years. Mean pre-operative BMI was 49.2 ± 7.8 kg/m^2^. Mean bodyweight was 143.6 ± 28.1 kg at first presentation and 141.9 ± 27.4 kg at surgery. 27.3% (*n* = 3896) of cases were diagnosed with T2D and 65.9% (*n* = 7233) with arterial hypertension (aHT). Mean age at reported diagnosis of obesity was 16 ± 11.0 years, with an average disease duration of 27.3 ± 12.0 years prior to surgery. SG is the most frequently performed procedure (47.2%, *n* = 6805) followed by RYGB (39.7%, *n* = 5722) and OAGB (13.1%, *n* = 1877). 89.7% (*n* = 12,927) of patients reported parental predisposition for obesity (maternal: 58.8%, paternal 45.7%, both 26.1%). Only 48.2% of patients lost weight pre-operatively, while 51.8% of patients showed no weight loss or even weight gain between first consultation and surgery. Patient characteristics are listed in Table 1.

**Table 1:** Pre-operative patient characteristics in responders versus non-responders and distribution of surgery types in these groups. No hypothesis tests were conducted at this level of comparison. *TWL* total weight loss, *T2D* type 2 diabetes, *aHT* arterial hypertension, *RYGB* Roux-en-y gastric bypass (RYGB), *SG* sleeve gastrectomy, *OAGB* one-anastomosis gastric bypass

	Total (*n* = /%)	1-year TWL < 20% (*n* = %)	1-year TWL > 20% (*n* = %)
Total	14,404	1119 (7.8%)	13,285 (92.2%)
Age (years)	43.9 ± 11.6	48.5 ± 11.8	43.3 ± 11.5
**Sex**			
Male	3756 (26.1%)	378 (33.8%)	3378 (25.4%)
Female	10,648 (73.9%)	741 (66.2%)	9907 (74.6%)
Disease onset of obesity (years)	15.5 ± 11.4	17.6 ± 13.1	15.4 ± 11.2
Obesity duration	28.3 ± 12.6	31.0 ± 13.5	28.1 ± 12.5
Paternal predisposition	5524 (45.7%)	433 (45.9%)	5091 (45.7%)
[unknown *n* (%)]	[*n* = 2323 (16.13%)]	[*n* = 228 (17.1%)]	[*n* = 2095 (16.03%)]
Maternal predisposition	7403 (58.8%)	560 (57.3%)	6843 (58.9%)
[unknown *n* (%)]	[*n* = 1812 (12.58%)]	[*n* = 185 (13.88%)]	[*n* = 1627 (12,45%)]
Pre-operative weight (kg) (Missing *n* = 5)	143.6 ± 28.1	139.3 ± 29.6	144.0 ± 27.9
Weight at surgery (kg)	141.9 ± 27.4	142.7 ± 30.1	141.8 ± 27.9
**Pre-operative weight change (kg)**			
= / > 0 kg	7459 (51.8%)	758 (67.7%)	6705 (50.5%)
< 0 kg	6945 (48.2%)	361(32.3%)	6580 (49.5%)
Pre-operative weight change (kg) (Missing *n* = 5)	− 1.8 ± 9.0	+ 3.4 ± 11.6	− 2.2 ± 8.6
Pre-operative BMI (kg/m^2^)	49.2 ± 7.8	49.2 ± 8.6	49.2 ± 7.7
T2D (Missing *n* = 130)	3896 (27.3%)	454 (40.6%)	3442 (25.9%)
Pre-operative HbA1c (%) (Missing *n* = 7089)	6.1 ± 1.2	6.5 ± 1.5	6.0 ± 1.2
aHT (Missing *n* = 3424)	7233 (65.9%)	663 (72.1%)	6570 (65.3%)
**Surgery type**			
RYGB	5722 (39.7%)	340 (30.4%)	5382 (40.5%)
SG	6805 (47.2%)	680 (60.8%)	6125 (46.1%)
OAGB	1877 (13.1%)	99 (8.8%)	1778 (13.4%)

**Fig. 2:**
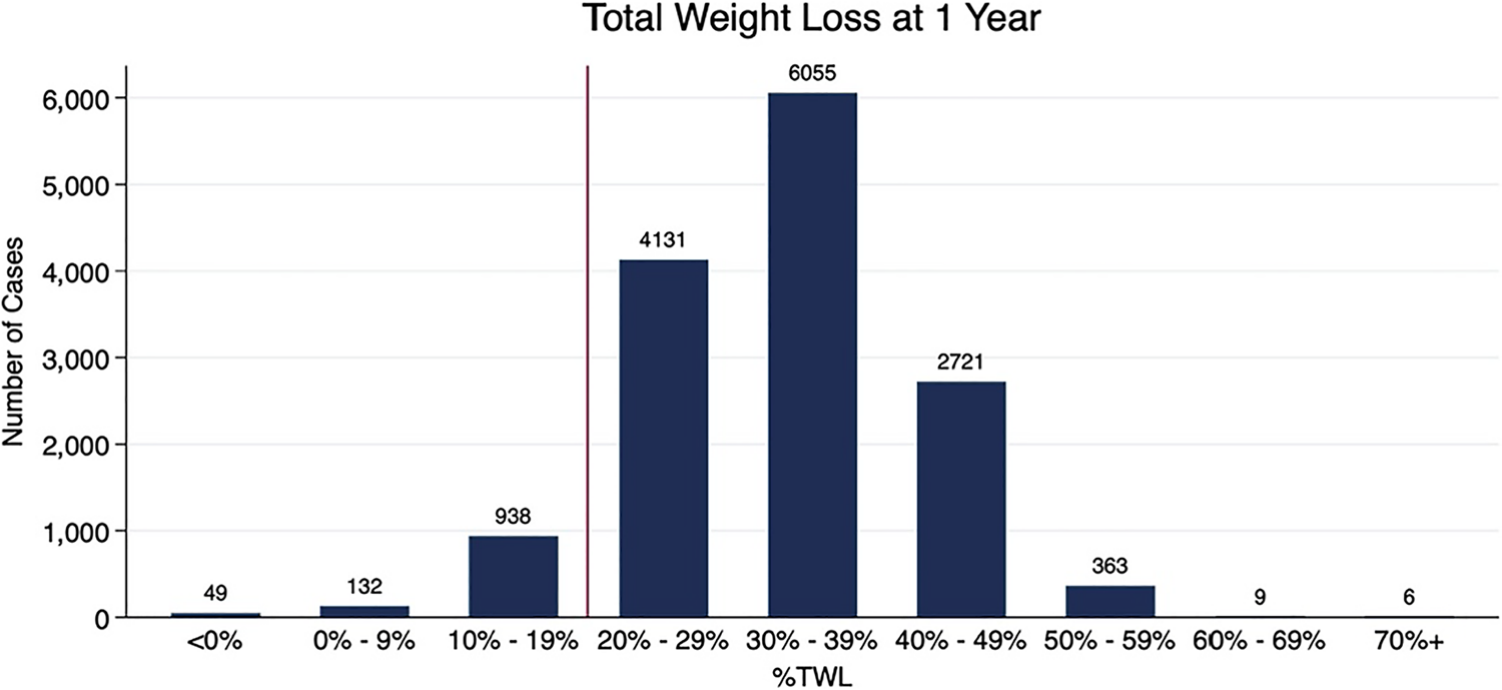
1-year TWL distribution of the StuDoQ | MBE study cohort. Boxes represent deciles and numbers above boxes indicate patient number. The vertical red line indicates the cut-off between responders and non-responders

Mean TWL is 32.7 ± 9.3% after 1 year and 32.2 ± 10.5% after 3 years. 7.8% (*n* = 1119) of patients show poor response at 1 year and 10.5% (*n* = 179) at 3 years. We illustrate TWL distribution in deciles in Fig. 2.

### Characteristics of Poor Response at 1 Year

Characteristics of successful and unsuccessful post-operative weight loss 1-year post-operatively are summarized in Table 1. At a descriptive level, surgical success was associated with younger age, female sex, earlier onset of disease, higher pre-operative weight, pre-operative weight loss, maternal predisposition, and inversely associated with T2D, aHT, and SG.

### Comparison of Surgery Subtypes

We describe distribution of mean pre-operative factors and outcome measures in RYGB, SG, and OAGB in Table 2. Patients undergoing SG had a higher pre-operative weight and BMI than RYGB and OAGB. TWL at 1 and 3 years as well as TWL failure show a clear gradient between SG versus RYGB and OAGB. The proportion of T2D patients was lowest in SG (23.6% vs 30.3% and 31.2% in RYGB and OAGB); this is reflected by mean HbA1c values. Prevalence of aHT was not relevantly different between groups.

**Table 2:** Patient characteristics and mean outcome measures by surgery type in the selected StuDoQ|MBE cohort. *SG* sleeve gastrectomy, *RYGB* Roux-en-y gastric bypass, *OAGB* one-anastomosis gastric bypass, *TWL* total weight loss, *T2D* type 2 diabetes, *aHT* arterial hypertension

	SG (*n* = 6805)	RYGB (*n* = 5722)	OAGB (*n* = 1877)
Age (years)	43.6 ± 11.9	44.0 ± 11.2	44.5 ± 11.6
**Sex**			
Male	1996 (29.3%)	1209 (21.1%)	551 (29.4%)
Female	4809 (70.7%)	4513 (78.9%)	1326 (70.6%)
Disease onset of obesity (years)	15 ± 11.8	15.9 ± 10.8	16.7 ± 11.2
Obesity duration (years)	28.6 ± 13	28.1 ± 12.2	27.9 ± 12.1
Paternal predisposition	2619 (45.8%)	2185 (46.4%)	720 (43.6%)
[unknown *n* = (%)]	[*n* = 1081 (15.9%)]	[*n* = 1017 (17.8%)]	[*n* = 225 (12%)]
Maternal predisposition	3515 (60.0%)	2916 (59.3%)	972 (56.7%)
[unknown *n* = (%)]	[*n* = 845 (12.4%)]	[*n* = 803 (14%)]	[*n* = 164 (8.74%)]
Weight at 1^st^ visit (kg)	148.0 ± 30.5	137.7 ± 24.0	145.9 ± 27.2
Weight at surgery (kg)	146.3 ± 29.8	135.9 ± 23.3	143.9 ± 26.3
BMI at surgery (kg/m^2^)	50.5 ± 8.4	47.5 ± 6.7	49.7 ± 7.6
Pre-operative weight change (kg)	− 1.7 ± 9.7	− 1.7 ± 8.2	+ 2.0 ± 8.5
Mean TWL at 1 year (%)	31.7 ± 9.6	33.4 ± 8.9	33.2 ± 9.0
Mean TWL at 3 years (%)	30.9 ± 11.0	33.0 ± 9.7	35.7 ± 10.4
1-year TWL < 20%	680 (10.0%)	340 (5.9%)	99 (5.2%)
3-year TWL < 20% (*n* = 1697)	112 (14.4%)	61 (7.7%)	6 (4.7%)
T2D	1589 (23.6%)	1726 (30.3%)	581 (31.2%)
HbA1c (%)	6.0 ± 1.1	6.1 ± 1.3	6.2 ± 1.3
1-year HbA1c (%)	5.9 ± 4.4	5.8 ± 4.6	5.6 ± 2.1
3-year HbA1c (%)	5.8 ± 2.9	5.6 ± 1.4	5.3 ± 0.6
aHT	3424 (66.5%)	2893 (65.0%)	916 (66.3%)

### Predictors of Surgical Failure

We explore crude associations of pre-operative demographics and clinical factors with non-response in univariate logistic regression analyses. The following features were found significantly associated with MBS failure: age (*p* < 0.001), pre-operative BMI kg/m^2^ (*p* < 0.001), male sex (*p* < 0.001), aHT (p < 0.001), T2D (*p* < 0.001), pre-operative weight loss/gain kg (*p* < 0.001), sleeve gastrectomy (*p* < 0.001), and onset of disease < 18 years of age (*p* < 0.001).

**Fig. 3:**
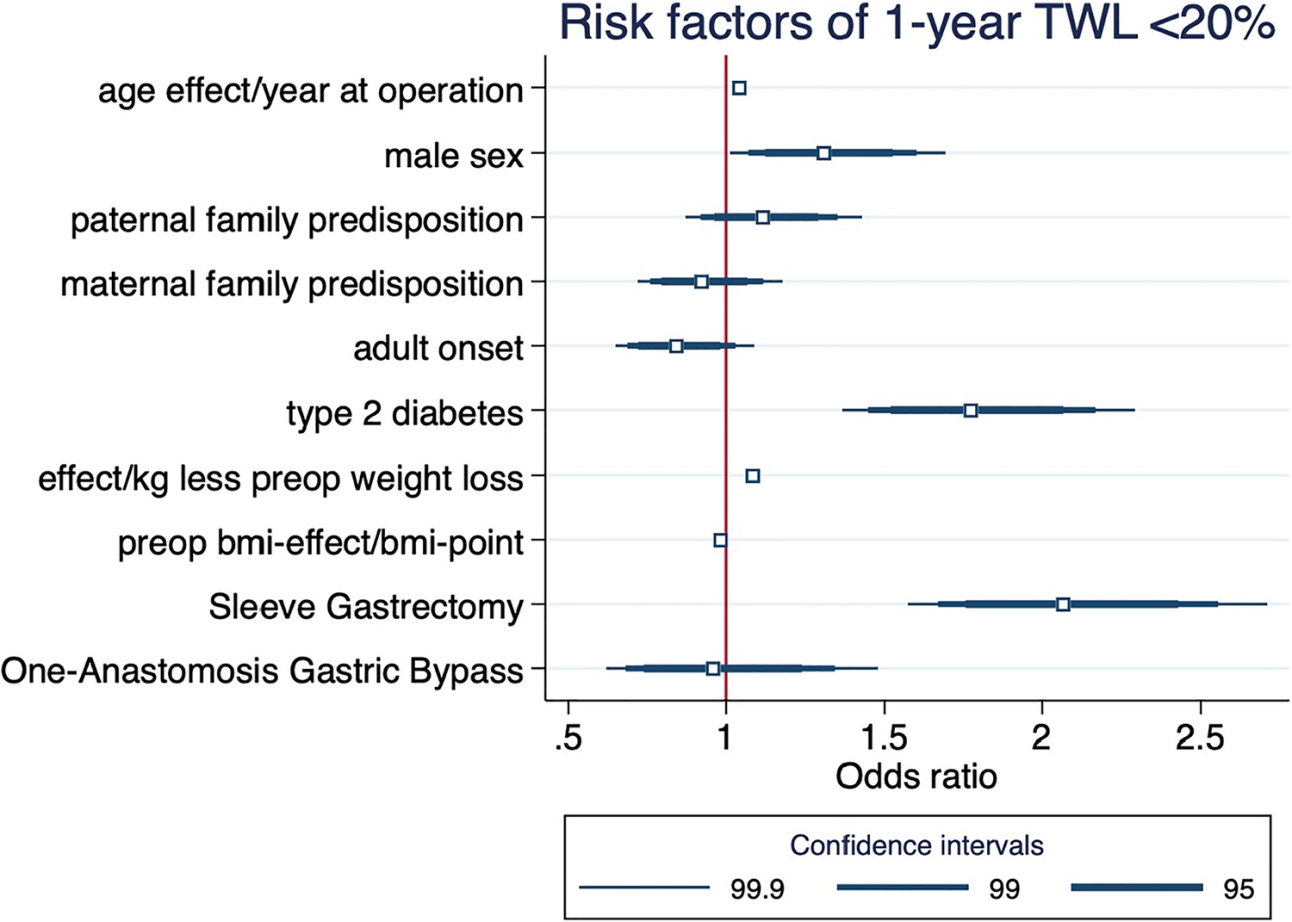
Adjusted odds ratio coefficients for risk factors associated with poor MBS response (1-year TWL < 20%). The estimated coefficients in this plot are derive from the multivariate logistic regression model described above. Bar thickness provides estimates of the 95, 99, and 99.9 confidence intervals. For age, pre-operative weight gain, pre-operative BMI, the odds ratio depicted is for a 1 unit increase of the respective covariate. “Adult-onset” indicates >  = 18 years. Roux-en-y gastric bypass is the reference category (OR = 1, not depicted) for sleeve gastrectomy and one-anastomosis gastric bypass

Fitting a multivariate logistic regression model, we examined covariates using forward and backward selection and included clinically relevant independent predictors, potential confounders, and modifiers of surgical failure in this register. aHT did not show an independent association with 1-year TWL failure. We excluded it from the final model due to higher proportion of missing values. 11,807 observations were included in the final multivariate model for whom complete data on these parameters were present. In this model, higher age (OR 1.04 per year, *p* < 0.001), poor pre-operative weight loss (OR 1.08 per kg, *p* < 0.001), T2D (OR 1.77, *p* < 0.001), male sex (OR 1.31 *p* = 0.004), and sleeve gastrectomy (OR 2.07, *p* < 0.001, RYGB = reference category) were associated with a significantly increased odds ratio (OR) for 1-year surgical failure. By contrast, pre-operative BMI (OR 0.98 per BMI point, *p* < 0.001) and adult onset of disease (OR 0.84, *p* = 0.028) were associated with a significantly decreased OR for surgical failure (Fig. 3). Paternal and maternal predisposition as well as anti-hypertensive medication use were not independently associated with surgical failure in this model but improved the discriminatory power of the model. Furthermore, we performed a post-estimation test according to Hosmer–Lemeshow to investigate goodness of fit. Applying 10 groups, the Hosmer–Lemeshow chi^2^ was 8.61 with a *p*-value of 0.38.

**Fig. 4:**
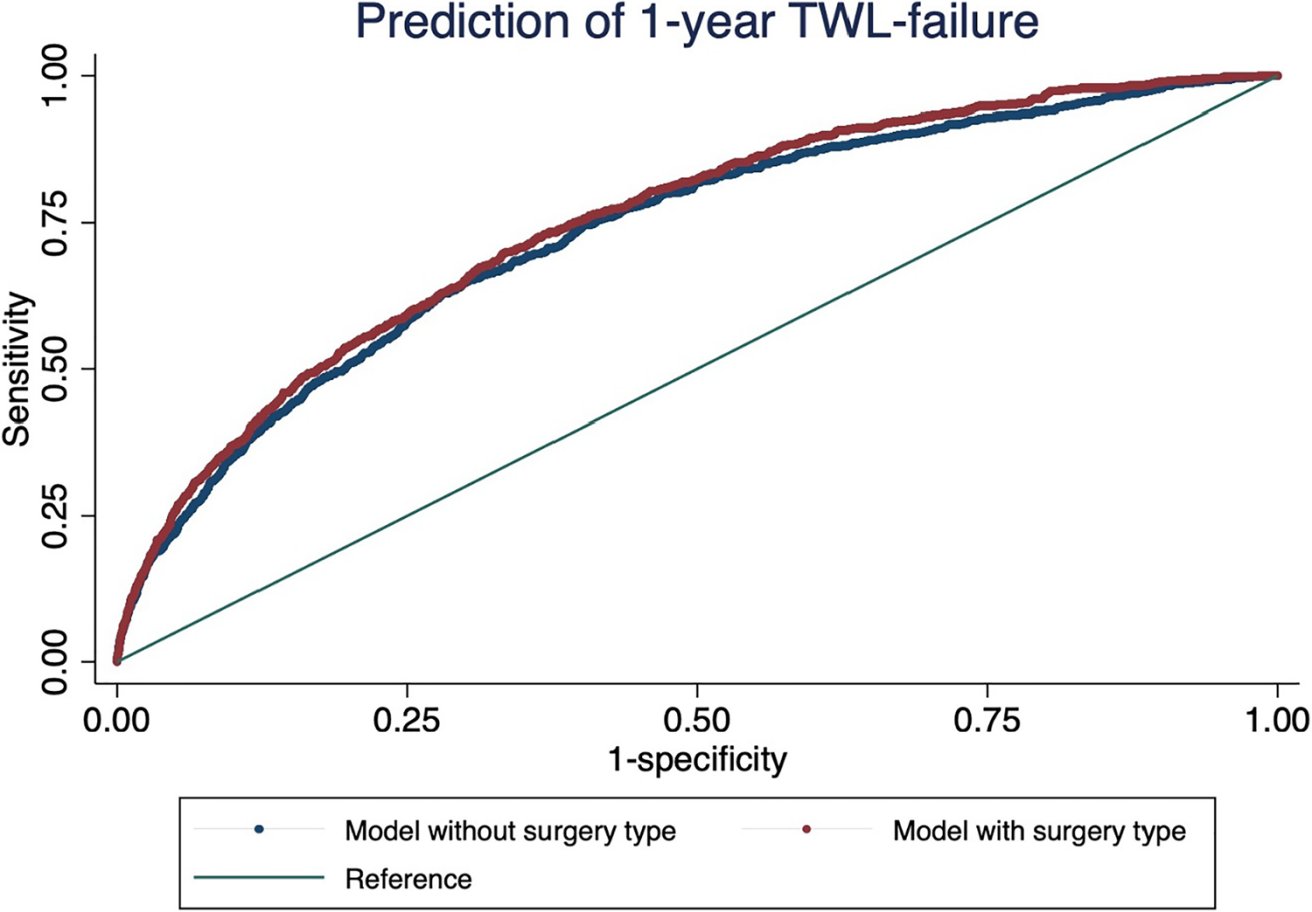
Comparison of two predictive models of 1-year TWL failure. We used the DeLong method to compare ROC-curves. The ROC curves show the predictive power of two models of 1-year TWL failure, one using all covariates (Fig. 3) without surgery type (blue curve) vs all covariates with surgery type (red curve). Adding surgery type independently improves the AUC from 0.732 to 0.749 (*p* < 0.001), which means that there is higher sensitivity when predicting surgical failure)

Because surgery type was associated with such a strong independent difference in the odds ratio for poor response, we used the DeLong method to compare ROC-curves of 2 multi-variate prediction models of surgical weight loss failure, one including surgery type as a pre-operative covariate, one without (Fig. 4). Adding surgery type as a covariate leads to a significant increase of the area under the curve (AUC) of 0.732 to 0.749 (chi^2^ = 18.11, *p* < 0.001). In this model surgery type significantly and independently contributes to the risk of non-response at 1 year.

## Discussion

A relevant proportion of patients respond poorly to MBS. Understanding risk factors associated with poor response as well as the underpinning mechanisms is necessary for individualising treatment. In this retrospective study of 14,404 German MBS patients, we investigate the association of parental predisposition for obesity and age at onset with 1-year TWL failure.

### Disease Duration and Heritability

We are currently investigating the association of parental predisposition for obesity and age at onset of disease in the StuDoQ|MBE cohort. We hypothesize that these factors are associated with patients’ response to surgery. In this MBS cohort, the proportion of patients who report paternal and maternal obesity is considerable (45.7% and 58.8%, respectively). Duration of disease prior to surgery is 28.3 years. We find that early onset of obesity is an independent predictor of 1-year TWL failure. Parental predisposition, however, is not independently associated with poor response. Parental predisposition did add to the discriminatory power of a multivariate model of surgical failure though. The contribution of heritable traits and parental predisposition to MBS response remains unclear and challenging to estimate [12–17]. These data indicate that patients with early onset of disease may be at higher risk of responding poorly to surgery. Early identification and individualized treatment could reduce the risk of poor surgical response.

### Pre-operative BMI and Weight Loss

Compared to other national registries (e.g., SOS trial), mean pre-operative BMI is significantly higher in this German cohort (49.8 kg/m^2^ vs 41.8 kg/m^2^), yet overall postoperative weight loss is comparable [18]. Higher pre-operative BMI values are associated with a lower odds ratio for TWL < 20%. Conversely, this implies that heavier patients are more likely to lose > 20% of their bodyweight post-operatively. Several investigations found higher baseline BMI positively associated with postoperative weight loss (6). This, however, is highly dependent on the outcome measurement used [4, 19, 20]. Proportional (TWL) and ratio measures (EWL) provide different estimates: heavier patients are more likely to have successful TWL but less successful EWL.

Additionally, in this cohort, there is a significant association of poor preoperative weight loss with non-response to MBS (OR = 1.03 per kg less weight loss, *p* < 0.001). This implies that a patient who gains 5 kg preoperatively has a 30% independently increased odds ratio of poor response compared to a patient who loses 5 kg preoperatively. Furthermore, preoperative weight loss has been associated with lower postoperative morbidity and mortality [21]. This result must be interpreted with caution, but it is at least plausible that unfavorable preoperative weight development may be associated with a higher risk for surgical failure. Current national guideless include professional dietary advice as a prerequisite for MBS to improve postoperative outcome.

### Type of Surgery

Our multivariate model provides a comparative effectiveness model of the most common bariatric procedures in terms of weight loss failure. More frequently, surgery types are compared regarding positive average endpoints. In this cohort, SG is independently associated with a 2.1-fold odds ratio for developing 1-year TWL failure. There was no superiority of RYGB over OAGB. Multiple studies have sought to estimate efficacy of different MBS procedures [21–24]. Two major trials have analyzed 5-year data on weight loss following bariatric surgery, comparing SG to RYGB [21, 22]. Despite no significant difference between the studies regarding weight loss, merged data between these two trials established a significant superiority of RYGB over SG in terms of excess weight loss (62.7% vs 55.5%) [24]. The YOMEGA trial, a recent multi-center RCT (*n* = 253) could not show inferiority of the OAGB compared to RYGB, regarding weight loss [25]. We believe that the results of our investigation add to these efficacy studies and complement them. If SG is independently associated with a relevantly increased risk of poor response, this may partly explain the difference of short-term and mid-term efficacy studies.

### Age

Age is an independent predictor of poor response. We have shown this in our study, and it is congruent with previous literature [26–28]. A precise estimate of age-attributable proportion of MBS response is difficult. A recent retrospective analysis of 1026 patients demonstrated higher excess BMI change and better remission of comorbidities in younger patients compared to patients over 60 years of age (T2D: OR 0.693, *p* = 0.038, aHT: OR 0.600 *p* = 0.024) [26]. A meta-analysis by Marczuk et al. showed higher morbidity of RYGB in elderly patients (OR 1.88, *p* = 0.03), with lower effectivity in terms of weight loss (*p* < 0.001) and remission of comorbidities (e.g., T2D: OR 0.64, *p* = 0.04, aHT: OR 0.33, *p* = 0.007) [27]. Overall, MBS can be performed safe and effectively in elderly patients, but morbidity, postoperative complications, and poorer weight loss are more frequently encountered compared to 30–39-year-old patients [28]. The significant impact of age and duration of disease on weight loss failure should be taken into account by clinicians that decide the timing and choice of MBS.

### Limitations

Due to the retrospective nature of this study, reporting bias may exist regarding parental predisposition and age at onset. Parental weight and metabolic health may fluctuate significantly throughout the years, and it is unknown which parental status is recalled and reported at time of data collection. Also, patients’ estimation of parental weight may be distorted due to a biased perception of “normal.” This is a nested cohort study to investigate the association between parental predisposition and age at onset with poor response. Our inclusion and exclusion criteria of parental predisposition and age of onset may lead to selection bias. This data, however, is provided by patients at random and there is no reason to assume a significant difference of covariate distribution from the rest of the register. Assuming there could be a variation, it would most likely be statistically insignificant due to the large cohort size. Endpoint estimation may be confounded by loss to follow-up.

Compliance at 1-year follow up in bariatric centers is high. After this time, the proportion of follow-up declines rapidly. It is unclear whether patients, who drop out of follow-up, are “responders” or “non-responders.” Studies estimate that removal from follow-up is associated with under-average outcome, suggesting surgical failure rates may, in reality, be higher [29, 30]. This reasoning also applies to data on 3-year follow up. Our data, however, shows a proportion of failure congruent with previous publications that report up to 11% TWL failure [3, 4, 6, 8, 31]. The StuDoQ|MBE register allows an “unknown” response to the question of parental predisposition. We regarded this as “missing.” This led to the exclusion of some observations from the complete case analysis and may bias results toward the null. We refrained from imputing “missing” data for the multivariate regression analysis, because we are uncertain if data is missing completely at random. Therefore, the number of observations analyzed in multivariate analysis is lower than the full cohort described. Despite a potential change in the effect size estimates, we do not believe that this would change the high significance of independent associations. The quality of the register’s data input is neither monitored nor validated, with many missing values. Other potential modifiers such as mental health or socioeconomic status were not included in this analysis, but may also have significant impact on surgical outcome.

## Conclusion

In a dynamic development of multi-generational chronic disease, our results provide current estimates of the distribution of parental predisposition for obesity and average age at onset in MBS patients. We find that the proportion of MBS patients in Germany reporting parental predisposition is very high and the average age at onset is under 15.5 years. Early onset of obesity, age, poor pre-operative weight loss, lower pre-operative BMI, SG, and T2D are independent predictors of surgical weight loss failure (TWL < 20%).
